# Right supernumerary kidney with urothelial carcinoma: A case report

**DOI:** 10.1097/MD.0000000000022329

**Published:** 2020-09-18

**Authors:** Xinghua Gao, Qingfei Xing, Xudong Luo, Tingshuai Cao, Shimin Zhang, Fei Yang, Daming Fan, Zhen Gao, Longyang Zhang, Feng Guo

**Affiliations:** a Department of Urology; b Department of Medical Imaging; c Department of Pathology, Central Hospital Affiliated to Shandong First Medical University, Jinan, Shandong Province, P.R. China.

**Keywords:** congenital anomaly, supernumerary kidney, urothelial carcinoma

## Abstract

**Rationale:**

A supernumerary kidney is an extremely rare renal anomaly. Currently, <100 cases are reported in the literature. There are only 2 right unilateral supernumerary kidneys reported in the literature thus far, but no confirmed cases of urothelial carcinoma in supernumerary kidneys. We report a case of a right supernumerary with urothelial carcinoma, which is, to the best of our knowledge, reported for the first time.

**Patient concerns:**

A 73-year-old female patient presented with intermittent, painless, whole course and gross hematuria for about 3 months. Her physical and laboratory examinations did not reveal any significant findings except positive occult blood in routine urine examination. Contrast-enhanced spiral computed tomography revealed a dysplastic supernumerary kidney under the normal right kidney.

**Diagnoses:**

The ureteroscopy showed that the ureter was Y-shaped in the middle part. The medial ureter led to a normal kidney. The lateral ureter was just 2 cm and led to a small cavity in which there was a mass whose biopsy showed urothelial carcinoma. The patient was subsequently diagnosed with a right supernumerary kidney with urothelial carcinoma.

**Intervention:**

Nephroureterectomy, including the right normal and supernumerary kidneys, and partial cystectomy by laparoscopy were performed after the ureteroscopy. The patient then received 6 cycles of gemcitabine and cisplatin regimen chemotherapy and regular intravesical epirubicin chemotherapy.

**Outcomes:**

No recurrence or metastasis was found on follow-up computed tomography performed 13 months postoperatively.

**Lessons:**

A supernumerary kidney is an extremely rare renal anomaly. Malignancy can occur in supernumerary kidneys.

## 1 Introduction

A supernumerary kidney is an extremely rare renal anomaly. It is defined as the third kidney (in addition to the two independent kidneys), with a distinct collecting system, blood supply, and well-defined capsule.^[1]^ Currently, <100 cases are reported in the literature, with the first case being reported in 1965.^[2]^ The real incidence of supernumerary kidneys cannot be calculated because of its unusual appearance. There are only 2 right supernumerary kidneys reported in the literature thus far.^[3,4]^ We report a case of a right supernumerary kidney with confirmed urothelial carcinoma, which is, to the best of our knowledge, being reported for the first time.

## 2 Case report

**Figure 1:**
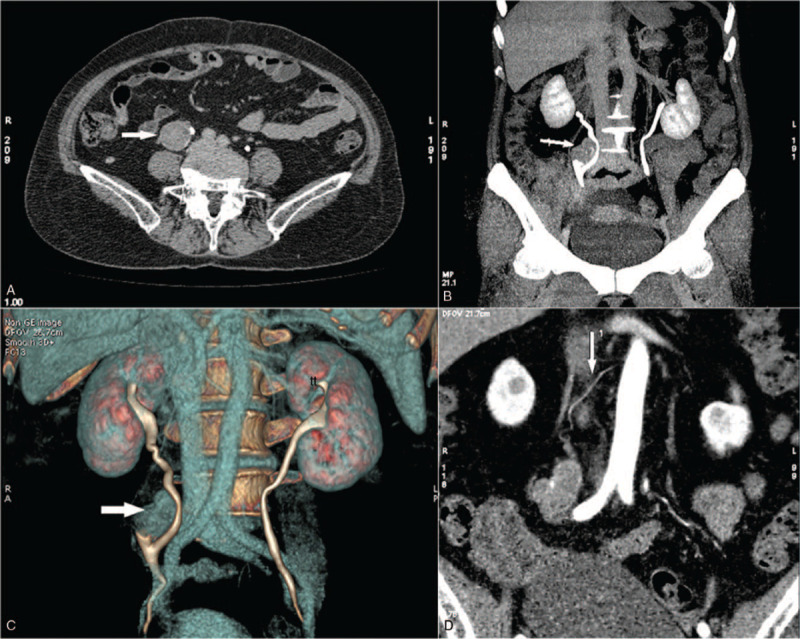
Computed tomography (CT) demonstrates a dysplastic supernumerary kidney (arrow) under the normal right kidney (A–C), and a separate arterial supply (arrow) originating from the aorta (D).

A 73-year-old female patient presented with intermittent, painless, whole course, and gross hematuria for approximately 3 months. The patient had a history of untreated hypertension. Her physical and laboratory examinations did not reveal any significant findings except positive occult blood in routine urine examination. Contrast-enhanced spiral computed tomography (CT) revealed a dysplastic supernumerary kidney measuring approximately 4.2 × 5.0 × 5.3 cm in size under the normal right kidney (Fig. 1A–C), which had a separate arterial supply originating from the aorta (Fig. 1D).

**Figure 2:**
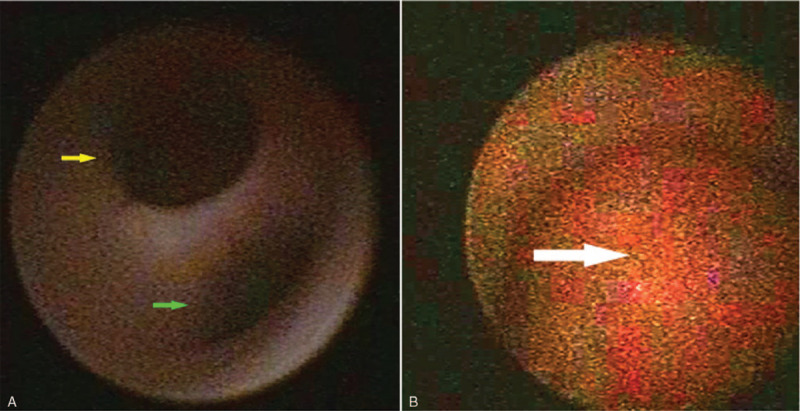
The ureteroscopy showing the right ureter with two branches in the middle part: the lateral ureter (yellow arrow) and medial ureter (green arrow) (A). The lateral ureter leads to a small cavity in which there was a mass (arrow) (B).

**Figure 3:**
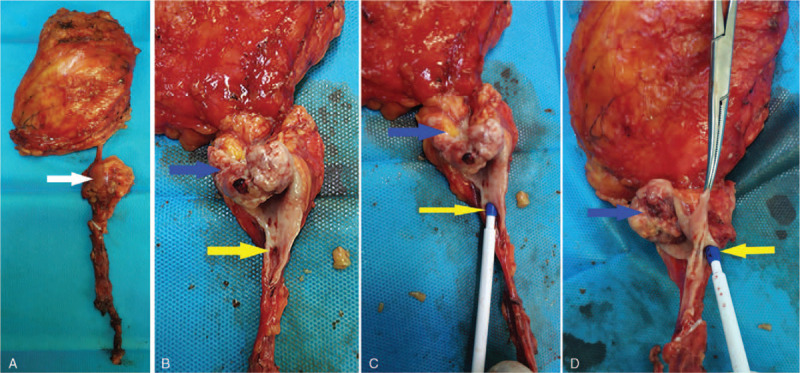
The postoperative specimen shows the supernumerary kidney (arrow) (A), tumor (blue arrow), and medial ureter leading to a normal kidney (yellow arrow) (B–D).

**Figure 4:**
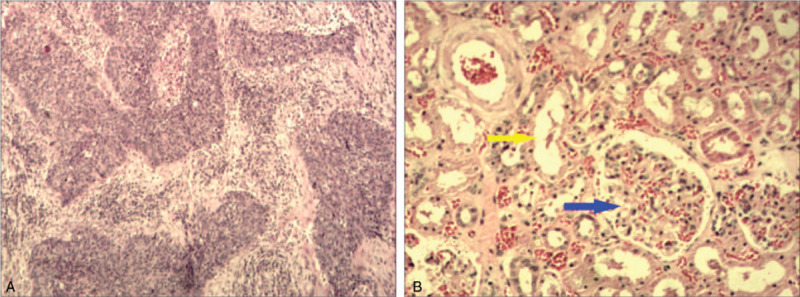
Postoperative pathology revealing high-grade urothelial carcinoma (hematoxylin and eosin staining, ×40) (A) and the glomerular (blue arrow) and tubular (yellow arrow) structures in the supernumerary kidney (hematoxylin and eosin staining, ×100) (B).

The ureteroscopy showed that the right ureter was Y-shaped in the middle part (Fig. 2A). The medial ureter (green arrow) led to a normal kidney. The lateral ureter (yellow arrow) was just about 2 cm and led to a small cavity in which there was a mass (Fig. 2B), whose biopsy showed urothelial carcinoma. Nephroureterectomy, including the right normal and supernumerary kidneys, and partial cystectomy by laparoscopy were performed after the ureteroscopy. The postoperative specimen showed 2 branches of the right ureter and a tumor in the supernumerary kidney (Fig. 3). Postoperative pathology revealed that the tumor was a high-grade urothelial carcinoma (Fig. 4A). Glomerular and tubular structures were found in the supernumerary kidney (Fig. 4B). The patient then received 6 cycles of gemcitabine and cisplatin regimen chemotherapy and regular intravesical epirubicin chemotherapy.

No recurrence or metastasis was found on follow-up CT performed 13 months postoperatively. There was no discomfort except for hair loss and occasional nausea. The patient was satisfied with the timely and effective treatment.

## 3 Discussion

A supernumerary kidney is a very rare congenital anomaly of the urinary tract. Only <100 case reports can be found in the literature. It is usually smaller than a normal kidney in terms of size and function.^[5]^ The supernumerary kidney can be either totally isolated from the ipsilateral kidney or attached to it through loose fibrous tissue.^[5]^ The supernumerary kidney is thought to result from an abnormal division of the nephrogenic cord into 2 separate metanephric blastemas at the fifth to seventh week of gestation, and it may have partially or completely duplicated ureters.^[6]^ Compared with a duplex kidney, a supernumerary kidney has a separate arterial supply originating from the aorta, venous drainage via the inferior vena cava, pelvicalyceal system, and distinct renal capsule.^[3,7,8]^ A supernumerary kidney is usually present on the left side. Although there are several bilateral supernumerary kidney reports, it is extremely rare to have a right unilateral supernumerary kidney, with only 2 cases were reported thus far.^[3,4,9]^

Some supernumerary kidney-associated congenital anomalies include horseshoe kidney malformations, ureteral atresia, imperforate anus, vaginal atresia, ectopic ureter implantation, urethral duplication, coarctation of the aorta, and meningomyelocele.^[10]^ Because of the hypoplastic nature of the involved renal element, urinary incontinence produced by ureteral ectopia from the supernumerary kidney is rarely seen.^[9]^ These anomalies are often asymptomatic and usually go undiagnosed until the fourth decade of life.^[10]^ Abdominal discomfort or a palpable mass, hypertension, and fever may be the most common presenting symptoms.^[3]^ A number of pathologic conditions, such as pyelonephritis, hydronephrosis, renal calculi, ureteropelvic junction obstruction, and benign and malignant neoplasms, may affect the supernumerary kidney.^[4]^ Carlson reported that 2 carcinomas had been seen in conjunction with a supernumerary kidney in 51 cases, without a definite pathological type.^[11]^ Exley and Hotchkiss reported a supernumerary kidney with clear cell carcinoma.^[12]^ The present case is the first confirmed urothelial carcinoma in a supernumerary kidney. It should be considered that malignancy can occur in supernumerary kidneys.

A supernumerary kidney with urothelial carcinoma can be treated as a renal pelvic carcinoma, undergoing a nephroureterectomy including the supernumerary and ipsilateral normal kidney and partial cystectomy by laparoscopy. Intravesical chemotherapy and platinum-based chemotherapy can be beneficial.

To conclude, the present case is more interesting in 3 respects. First, this is the third right unilateral supernumerary kidney ever reported. Second, this is the first supernumerary kidney with confirmed urothelial carcinoma. Third, the combination of radical surgery and chemotherapy is safe and effective for this patient.

## 4 Consent for publication

Informed written consent was obtained from the patient for publication of this case report. The presented data are anonymized, and the risk of identification is minimal.

## Author contributions

**Conceptualization:** Xinghua Gao.

**Formal analysis:** Tingshuai Cao, Shimin Zhang.

**Pathological recognition:** Fei Yang, Daming Fan.

**Image recognition:** Xudong Luo, Zhen Gao.

**Writing – original draft:** Xinghua Gao, Qingfei Xing.

**Writing – review & editing:** Longyang Zhang, Feng Guo.

## Abbreviations

Abbreviation: CT = computed tomography.

## References

[1] Patel R Singh H Willens D. Bilateral supernumerary kidneys: how much is too much? BMJ Case Rep 2014;2014:bcr2013202677. 10.1136/bcr-2013-202677 PMC3987526 24692375

[2] Sureka B Mittal MK Mittal A. Supernumerary kidneys—a rare anatomic variant. Surg Radiol Anat 2014;36:199 202. 23670608 10.1007/s00276-013-1135-z

[3] Kumar M Kumar G Barwal K. Right supernumerary kidney: a rare entity. Urol Case Rep 2019;23:97 8. 30729095 10.1016/j.eucr.2019.01.001 PMC6352298

[4] Al Dandan O Hassan A Almutairi A. Malrotated right supernumerary kidney: case report of a rare anomaly. Urol Case Rep 2019;26:100966. 31360639 10.1016/j.eucr.2019.100966 PMC6639741

[5] Ramanathan S Kumar D Khanna M. Multi-modality imaging review of congenital abnormalities of kidney and upper urinary tract. World J Radiol 2016;8:132 41. 26981222 10.4329/wjr.v8.i2.132 PMC4770175

[6] N’Guessan G Stephens FD. Supernumerary kidney. J Urol 1983;130:649 53. 6887391 10.1016/s0022-5347(17)51385-3

[7] Maranhão C Miranda C Santos C. Congenital upper urinary tract abnormalities: new images of the same diseases. Radiol Bras 2013;46:43 50.

[8] Favorito LA Morais AR. Evaluation of supernumerary kidney with fusion using magnetic resonance image. Int Braz J Urol 2012;38:428 9. 22765860 10.1590/s1677-55382012000300018

[9] Tada Y Kokado Y Hashinaka Y. Free supernumerary kidney: a case report and review. J Urol 1981;126:231 2. 7265371 10.1016/s0022-5347(17)54457-2

[10] Mejia M Limback J Ramirez A. A case of supernumerary kidney. Cureus 2018;10:e3686. 30761238 10.7759/cureus.3686 PMC6368426

[11] Carlson HE. Supernumerary kidney: a summary of 51 reported cases. J Urol 1950;64:224 9. 15429184 10.1016/S0022-5347(17)68623-3

[12] Exley M Hotchkiss W. Supernumerary kidney with clear cell carcinoma. J Urol 1944;51:569 78.

